# Social isolation and loneliness prevention among rural older adults aging-in-place: a needs assessment

**DOI:** 10.3389/fpubh.2024.1404869

**Published:** 2024-10-15

**Authors:** Jodi L. Southerland, Shimin Zheng, Kayla Dodson, Erin Mauck, Juanita-Dawne R. Bacsu, Monique J. Brown, Jeremy Holloway, Steffi M. Kim, Ayse Malatyali, Matthew Lee Smith

**Affiliations:** ^1^Department of Community and Behavioral Health, East Tennessee State University, Johnson City, TN, United States; ^2^Department of Epidemiology and Biostatistics, East Tennessee State University, Johnson City, TN, United States; ^3^School of Nursing, Thompson Rivers University, Kamloops, BC, Canada; ^4^Department of Epidemiology and Biostatistics, Arnold School of Public Health, University of South Carolina, Columbia, SC, United States; ^5^Department of Geriatrics, University of North Dakota School of Medicine and Health Sciences, Grand Forks, ND, United States; ^6^Department of Psychology, University of Alaska, Anchorage, AK, United States; ^7^College of Nursing, University of Central Florida, Orlanda, FL, United States; ^8^Department of Health Behavior, Center for Community Health and Aging, Texas A&M University, College Station, TX, United States

**Keywords:** social isolation, loneliness, rural, older adults, aging-in-place

## Abstract

**Introduction:**

The adverse effects of social isolation and loneliness (SI/L) have been documented among older adults in rural communities and contribute to poor health outcomes, premature disability and mortality, and increased burden on the healthcare system. The identification of factors contributing to SI/L among older adults can build the foundation for rural policymakers and leaders to allocate resources and develop tailored strategies more efficiently. The purpose of this article is to describe findings from a needs assessment designed to understand local factors that contribute to SI/L among rural older adults in a county in Northeast Tennessee. Findings from the needs assessment will be used by local stakeholders to develop strategies to promote age-friendly initiatives.

**Methods:**

Eighty-two older adults [ages 62 to 74 years (59%); non-Hispanic white (95%); female (71%)] from three senior apartment complexes in a Northeast Tennessee county completed an 87-item needs assessment survey. The evaluation of social isolation utilized Lubben’s 6-item Social Network Scale, while loneliness was assessed using the 3-item UCLA Loneliness Scale. Logistic regression analysis was used to identify predictors of SI/L. Given the limited sample size, statistical significance was considered at *p* < 0.10.

**Results:**

The prevalence of social isolation and loneliness was 42% and 37%, respectively. Residing in the county <5 years [Adjusted OR (AOR): 3.35; 95% CI: 1.04–10.81; *p* = 0.04] and reporting resource-related barriers to aging-in-place (AOR: 6.56; 95% CI: 2.00–21.57; *p* = 0.004) were associated with increases in the odds of social isolation; whereas interest in intergenerational activities decreased the odds of social isolation (AOR: 0.19; 95% CI: 0.05–0.69; *p* = 0.01). Boredom (AOR: 4.06; 95% CI: 1.63–12.11; *p* = 0.01) and limited knowledge about community services (AOR: 4.61; 95% CI: 1.42–15.02; *p* = 0.01) quadrupled the odds of loneliness. Similarly, older adults who were frail (AOR: 2.69; 95% CI: 0.88–8.17; *p* = 0.08) and who rated their community livability as low (AOR: 3.35; 95% CI: 0.81–13.87; *p* = 0.09) were more likely to experience loneliness.

**Discussion:**

This needs assessment provided important information about the individual and social drivers of SI/L among rural older adults in the community. Findings support the generation of localized data to support muti-partner efforts to design sustainable programs to address SI/L.

## 1 Introduction

The adverse effects of social isolation and loneliness (SI/L) have been documented among rural older adults and contribute to poor health outcomes, premature disability and mortality, and increased burden on the healthcare system (1–3). Older adults generally, and those in rural Appalachia specifically, rely on informal networks of family and friends to provide social support (4). However, many of these individuals experience structural barriers (e.g., geographic isolation, inadequate transportation services, sparse populations, and limited internet access) to connecting with family, friends, and neighbors (4–6). In addition, the Appalachian region is characterized as having multiple layers of vulnerability, such as a high proportion of older adults, limited availability of health and social services, and distinct cultural values that may inhibit health-seeking behaviors, a context referred to as “triple jeopardy” (4). Thus, older adults who tend to have more health problems and live in these rural areas face more challenges in accessing health and social services than their urban counterparts (7–9). These socio-structural barriers can reinforce existing health inequities, resulting in heightened vulnerability to SI/L.

Identifying factors that contribute to older adults’ SI/L can help rural policymakers and organizations target their resources and programming more effectively. However, the lack of local data is an ongoing challenge in these rural Appalachian communities (10, 11). Grassroots efforts may hold promise to address rural data gaps by generating local data, which can deepen stakeholders’ insights and strengthen efforts to implement specific, action-oriented solutions. This needs assessment reports on a local initiative to identify factors associated with SI/L among rural older adults aging-in-place in a Central Appalachian county within Northeast Tennessee.

## 2 Background and rationale

### 2.1 Social isolation and loneliness

SI/L have reached epidemic proportions in the U.S. (12). Although SI/L represent distinct concepts, they are often used interchangeably in the literature (1). While social isolation refers to an objective state of having few social relationships or infrequent social contact with others, loneliness reflects the individual’s subjective dissatisfaction with the frequency and closeness of relationships despite the actual interactions with others (3).

SI/L are associated with some similar and distinct negative health outcomes. These include heart disease, depression, anxiety, suicidality, dementia, frailty, nursing home admission, and premature disability and death (3, 13, 14). Studies estimate that SI/L are associated with a 26–29% increased risk of all-cause mortality (1). Recent research indicates that social isolation is a stronger predictor of all-cause mortality, while loneliness is more strongly associated with poor psychological well-being (2). Annually, social isolation results in $6.7 billion in additional Medicare spending (15).

Roughly 25% of older adults (ages 65 years and older) in the United States are socially isolated and 43% of adults ages 60 years and older feel lonely (3). Older adults are at increased risk of SI/L due to predisposing factors such as advanced age, lower educational attainment, living alone, chronic conditions, functional and cognitive impairment, smaller social networks, loss of family or friends, and retirement, among others (3). Some rural older adults have an even higher risk because they are more likely to live alone than their urban counterparts (16) and may not see or even communicate with another person for days at a time. Older adults in rural Appalachia may have greater susceptibility to the adverse health and psychological outcomes associated with SI/L due to underlying poor health status (7, 17) and limited access to behavioral and mental health services (4). Thus, it is important to take these factors into consideration when addressing SI/L in rural Appalachia (18).

### 2.2 Aging-in-place in Appalachia

Tennessee is home to more than 1.6 million residents over age 60 (19). Based on reports from America’s Health Rankings, Tennessee consistently falls in the bottom quartile of states for health outcomes among older adults over age 65 (20). Notable challenges include disease burden, limited access to clinical care and supportive social services, and risk of social isolation (20). In Tennessee, 52 out of 95 counties are in Central Appalachia. This region of Appalachia is predominately non-Hispanic White (21) and has experienced the largest growth in the older adult population in the region (22).

The Appalachian region is disproportionately burdened with poor social, economic, and health-related outcomes (22). Among 41 health indicators, the Appalachian region lags behind the U.S. on 33 of them, including seven of the 10 leading causes of death (7). Rural parts of the region have even greater risk for premature disability and mortality than urban areas (7). In this article, “rural” is defined as sparsely populated areas lying outside of urban centers (23) as defined by the United States Department of Agriculture Rural–Urban Commuting Area Codes (24). Many of the rural communities lack the infrastructure and resources to support healthy longevity (5, 7, 22). Consequently, rural older adults living in Appalachia often experience unique challenges to aging-in-place, which refers to having the ability to live safely and independently in one’s own home and community for as long as one chooses to do so (25). Rural older adults in these communities face a number of obstacles to social interactions, such as geographic isolation, environments that are not always walkable or socially conducive, a lack of economic resources, and restricted access to cellular and broadband Internet. Rural older adults are also more vulnerable to health problems because they are typically less mobile than their younger counterparts and more dependent on resources specific to their local community, creating difficulty in accessing mental health services among other medical services (26–28).

## 3 Context and essential elements of the needs assessment

Older adults are the fastest-growing age group in Tennessee. According to the Boyd Center for Business and Economic Research at the University of Tennessee, the proportion of individuals 65 and older will increase by almost 40% between 2020 and 2040 (29). Northeast Tennessee is aging even more rapidly than the rest of the state. Older adults in Northeast Tennessee have a higher burden of disease and disability compared to statewide averages. This is alarming because the patient-to-provider ratio in the eastern part of the state is 2.75 times higher than that in other parts of Tennessee. Older residents in Northeast Tennessee are also more likely to experience financial vulnerability, due in part to low educational attainment and a high number of grandparents raising grandchildren (19, 30). These cumulative disadvantages contribute to widening health inequities, particularly in persistently poor counties.

Given the significant risk for poor health outcomes among older Tennesseans, multi-partner efforts are needed to engage the local community in addressing key issues that impact this demographic (31). Multi-partner, grassroots initiatives are important in rural communities because they build on the existing infrastructure to foster collaboration, share resources, and address local concerns (32). This needs assessment builds on the efforts of a network of local service organizations working to promote healthy aging in counties within Northeast Tennessee. Because leaders in the network are familiar with the unique strengths and challenges of the community, they can more readily address emerging issues such as SI/L.

The needs assessment was designed to identify factors associated with SI/L and strategies to strengthen social connections among older adults. Network members selected one county as the needs assessment site because efforts were already underway to address the unmet needs of older adults. This county ranks in the bottom 50% of all counties in the state for health outcomes, health behaviors, and social and economic indicators (33). The county is also classified as rural (24) and is designated a health professional shortage area (34). The purpose of this article is to describe findings from the needs assessment related to the factors associated with SI/L.

## 4 Materials and methods

### 4.1 Needs assessment procedures

We conducted a needs assessment survey of older adults using purposive sampling in three affordable housing apartment complexes in a county within Northeast Tennessee between February and March 2023. This project was reviewed by the Institutional Review Board (IRB) at East Tennessee State University and deemed non-human subjects research because it was a needs assessment conducted for multi-stakeholder planning purposes to inform age-friendly initiatives. Participants were recruited via flyers distributed to residents of the apartments either in person or via mail. Residents were eligible to complete the survey if they were ages 62 years and older and spoke English. On the scheduled dates, two needs assessment team members returned to the apartments. While the university IRB deemed this needs assessment as non-human subjects research, our team took extra precautions to ensure respect and ethical considerations were addressed. During the resident engagement process, team members described the purpose of the needs assessment and received verbal consent from individuals prior to distributing the self-administered surveys. Only those who agreed to participate were given surveys to complete. Twelve individuals declined to participate in the needs assessment, and one individual did not meet the eligibility criteria. A total of 82 older adults completed the survey which took approximately 30 minutes to complete. Participants received $50 upon completion of the survey.

### 4.2 Measures

The 67-item survey was organized into the following seven thematic sections: general daily life; health status; socialization/recreation; relationships with others; neighborhood characteristics; programs and services for older adults; and sociodemographic characteristics.

#### 4.2.1 Outcome variables

Two outcome variables were included in the analysis. Social isolation was measured using Lubben’s 6-item Social Network Sale (LSNS-6) (35). The LSNS-6 is a validated objective measure of social network size, including the number and frequency of contact with family and friends. Items are scored from 0 to 5 and summed to provide a total score ranging from 0 (higher risk) to 30 (lower risk). Risk of social isolation was defined as a score below 12 (35). For the present analysis, the Cronbach’s alpha was 0.87. Loneliness was measured with the 3-item UCLA Loneliness Scale, which measures three subjective aspects of loneliness: lack of companionship, feeling left out, and feeling isolated from others (36). There were three response options: 1 = hardly ever, 2 = some of the time, and 3 = often. Scores range from 3 to 9, with loneliness defined as having a composite score of 6 or more (37). Cronbach’s alpha was 0.87 in the present analysis.

#### 4.2.2 Explanatory variables

We included 17 explanatory variables in the analysis. The selection of these variables was either well-established risk factors associated with SI/L, such as individual, neighborhood, and socio-cultural characteristics (3) or variables of interest to the researchers. The variables of interest to the researchers (e.g., interest in having more friends, activities, or intergenerational connections, knowledge about community services, and length of residence) were selected because of their potential connection to SI/L.

##### 4.2.2.1 Neighborhood conditions

Resource-related barriers to aging-in-place were measured with three items: In the last 12 months have you: (1) put off going to the doctor because of transportation; (2) needed to see a doctor but could not because of cost; and (3) had trouble pay bills (utilities, phone, medicine) due to cost (1 = yes, 0 = no). Experiencing resource-related barriers to aging-in-place was defined as responding *yes* to all three items.

One item was used to measure the level of knowledge about community services. Participants rated their knowledge on a 4-point Likert scale, from 1 = very informed to 4 = no, I do not feel informed. Low levels of knowledge were defined as responding *somewhat informed* or *no, I do not feel informed*.

Community livability was assessed with two items. Participants indicated whether their community was safe (1 = yes, 0 = no) and a good place for people to live as they age (4-point Likert scale: 1 = excellent to 0 = poor). Low community livability was defined as responding *no* to the first item and *fair* or *poor* to the second item.

##### 4.2.2.2 Interpersonal relationships and activities

Boredom, wanting more friends, wanting to participate in more activities, and interest in intergenerational activities (e.g., participating in activities with youth or young adults) were assessed with one item each (1 = yes, 0 = no).

Eighteen items were used to measure participants’ engagement in the following leisure time activities (38): (1) shop; (2) visit with family or friends in person; (3) visit with your neighbors in person; (4) exercise [walking, running, weights, etc]; (5) participate in a club or civic group; (6) go to church, Bible studies, prayer; (7) talk on the phone with family or friends; (8) talk on video call/internet with family or friends; (9) exchange text messages with family or friends; (10) provide help to family, friends or neighbors; (11) participate in activities at the senior center; (12) care for a pet; (13) do housework or home maintenance; (14) participate in a hobby alone in your house; (15) participate in hobbies with groups; (16) attend movies, sports, or community events; (17) volunteer or help in the community; and (18) use a computer iPad, tablet, or smartphone. Low leisure time activity was defined as responding *rarely* or *never* to more than nine items.

##### 4.2.2.3 Frailty

We used 10 questions to assess frailty (39, 40). Frailty was defined as a *yes* response to six or more items assessing physical health and functional status: (1) 4 or more chronic conditions derived from the question “has a doctor ever told you that you have any of the following conditions? (check all that apply)” (41) [21-item list of common conditions affecting older adults]; (2) disability defined as having a disability or health problem that limits the individual from participating in activities (e.g., housework, driving, recreation) (42); (3) pain defined as having pain that interfered somewhat to very much with daily activities in the past 7 days (43); (4) polypharmacy defined as taking five or more medications daily (44); (5) limitations to activities of daily living defined as difficulty performing activities such as walking, bathing, dressing in the last 12 months (45); (6) limitations to instrumental activities of daily living defined as difficulty doing routine activities such as household chores or shopping in the past 12 months (45) (7) falls risk defined as having fallen or injured yourself in your home in the past 3 months (46); (8) emergency department or urgent care use defined as having used the emergency room or urgent care in the past 3 months (47); (9) currently uses home health services (48); (10) and needs or uses durable medical equipment (e.g., walkers, wheelchairs, lift chairs) (49).

#### 4.2.3 Sociodemographic characteristics

Measured demographic characteristics included age, gender, race, education, marital status, number of people living in the household, annual income, education, and length of residence in the county. Age was recoded as a categorical variable (<80 and ≥ 80 years) because individuals 80 and older are at greatest risk for social isolation (50).

### 4.3 Analysis

We conducted bivariate analysis to identify potential risk factors associated with SI/L. Variables with a *p*-value of ≤0.20 (51) in the simple logistic regression models were included in multiple logistic models using stepwise-backward selection procedures. Through this process, variables were systematically eliminated based on their *p*-values until those remaining in the model had a significance level of 10% (52, 53). Variance inflation factors (VIF) were computed for each multiple logistic regression model to assess multicollinearity, and no evidence of multicollinearity between risk factors was found (all VIFs <1.20). The analysis used SAS (SAS 9.4, SAS Institute, Inc., Cary, NC).

## 5 Results

### 5.1 Descriptive statistics

The descriptive statistics are presented in Table 1. The needs assessment included 82 participants. Roughly 18% of the participants were ages 80 years and older. The majority were non-Hispanic white (95.1%), and nearly three-fourths (70.7%) were female. A little more than two-thirds (69.5%) reported incomes of $24,000 or less or had less than a high school degree or graduated from high school. Most participants lived in the county for 5 years or longer (75.6%), were unmarried (e.g., single, widowed, divorced) (92.6%), and lived alone (91.5%). One-in-three (37.8%) were frail.

**Table 1:** Descriptive statistics for the sociodemographic characteristics, neighborhood conditions, and interpersonal relationships and activities.

	Isolated *n* (%)	Not isolated *n* (%)	Lonely *n* (%)	Not lonely *n* (%)
	34 (41.5)	48 (58.5)	30 (36.6)	52 (63.4)
Age				
≥ 80 years	4 (11.8)	11 (22.9)	6 (20.0)	9 (17.3)
< 80 years	30 (88.2)	37 (77.1)	24 (80.0)	43 (82.7)
Gender				
Female	21 (61.8)	37 (77.1)	20 (66.7)	38 (73.1)
Male	13 (38.2)	11 (22.9)	10 (33.3)	14 (26.9)
Race/Ethnicity				
Non-Hispanic Whites	33 (97.1)	45 (93.8)	28 (93.3)	50 (96.2)
Minorities	1 (2.9)	3 (6.2)	2 (6.7)	2 (3.8)
Annual income				
≤ $24,000	24 (70.6)	33 (68.8)	22 (73.3)	35 (67.3)
> $24,000	10 (29.4)	15 (31.2)	8 (26.7)	17 (32.7)
Education				
High school graduate or less	24 (70.6)	33 (68.8)	21 (70.0)	36 (69.2)
Some college or college degree	10 (29.4)	15 (31.2)	9 (30.0)	16 (30.8)
Marital status				
Not married (single, widowed, divorced)	31 (91.2)	45 (93.8)	28 (93.3)	48 (92.3)
Married or lives with partner	3 (8.8)	3 (6.2)	2 (6.7)	4 (7.7)
Length of residence				
< 5 years	11 (32.4)	9 (18.8)	6 (20.0)	14 (26.9)
≥ 5 years	23 (67.6)	39 (81.2)	24 (80.0)	38 (73.1)
Lives alone				
Yes	32 (94.1)	43 (89.6)	27 (90.0)	48 (92.3)
No	2 (5.9)	5 (10.4)	3 (10.0)	4 (7.7)
Resource-related barriers to aging-in-place				
Yes	15 (44.1)	8 (16.7)	14 (46.7)	9 (17.3)
No	19 (55.9)	40 (83.3)	16 (53.3)	43 (82.7)
Frailty				
Yes	17 (50.0)	14 (29.2)	16 (53.3)	15 (28.8)
No	17 (50.0)	34 (70.8)	14 (46.7)	37 (71.2)
Boredom				
Yes	19 (55.9)	19 (39.6)	20 (66.7)	18 (34.6)
No	15 (44.1)	29 (60.4)	10 (33.3)	34 (65.4)
Leisure time activities				
Low	17 (50.0)	16 (33.3)	15 (50.0)	18 (34.6)
High	17 (50.0)	32 (66.7)	15 (50.0)	34 (65.4)
Wants more friends				
Yes	21 (61.8)	25 (52.1)	21 (70.0)	25 (48.1)
No	13 (38.2)	23 (47.9)	9 (30.0)	27 (51.9)
Wants to participate in more activities				
Yes	20 (58.8)	23 (47.9)	14 (46.7)	29 (55.8)
No	14 (41.2)	25 (52.1)	16 (53.3)	23 (44.2)
Livable community				
Low	9 (26.5)	6 (12.5)	10 (33.3)	5 (9.6)
High	25 (73.5)	42 (87.5)	20 (66.7)	47 (90.4)
Knowledge about community services				
Low	24 (70.6)	21 (43.8)	24 (80.0)	21 (40.4)
High	10 (29.4)	27 (56.2)	6 (20.0)	31 (59.6)
Interest in intergenerational activities				
Yes	6 (17.6)	17 (35.4)	11 (36.7)	12 (23.1)
No	28 (82.4)	31 (64.6)	19 (63.3)	40 (76.9)

As for neighborhood conditions, most participants rated the community livability as high (81.7%); whereas one-quarter reported resource-related barriers to aging-in-place (28.0%) and over half reported low levels of knowledge about community services (54.9%). Regarding interpersonal relationships and activities, almost half reported boredom (46.3%) and four-in-ten wanted to participate in more activities (40.2%). Although more than half of participants wanted more friends (56.1%), only one-quarter of participants were interested in opportunities for intergenerational engagement (28.0%).

### 5.2 Prevalence of social isolation and loneliness

Four-in-ten participants were socially isolated (41.5%) and over one-third of participants were lonely (36.6%).

### 5.3 Risk factors for social isolation and loneliness

Table 2 presents a summary of the outcomes from both simple and multiple logistic regression analyses conducted to identify predictors of social isolation and loneliness.

**Table 2:** Univariate and multivariate analyses to determine factors independently associated with social isolation and loneliness.

Variables	Social isolation using Lubben social network scale				Loneliness using the UCLA Loneliness scale			
	Crude OR (CI)	*p*- value	Adjusted OR (CI)	*p*- value	Crude OR (CI)	*p*- value	Adjusted OR (CI)	*p*- value
Age (≥80 years vs. <80 years)	0.45 (0.13–1.55)	0.21			1.19 (0.38–3.76)	0.76		
Gender (male vs. female)	2.08 (0.79–5.47)	0.14			1.36 (0.51–3.60)	0.54		
Race (non-Hispanic white vs. minority)	2.17 (0.22–20.00)	0.50			0.56 (0.07–4.17)	0.57		
Annual income (≤$24,000 vs. >$24,000)	1.09 (0.42–2.84)	0.86			1.34 (0.49–3.61)	0.57		
Education (high school graduate or less vs. some college/college degree)	1.09 (0.42–2.84)	0.86			1.04 (0.39–2.76)	0.94		
Marital status (not married vs. married or lives with partner)	0.69 (0.13–3.64)	0.66			1.17 (0.20–6.78)	0.86		
Length of residence (<5 years vs. ≥5 years)	2.07 (0.74–5.75)	0.16	3.35 (1.04–10.81)	0.04^**^	0.68 (0.23–2.01)	0.48		
Lives alone (yes vs. no)	1.86 (0.34–10.21)	0.48			0.75 (0.16–3.60)	0.72		
Resource-related barriers to aging-in-place (yes vs. no)	3.95 (1.43–10.91)	0.01	6.56 (2.00–21.57)	0.002^***^	4.18 (1.52–11.54)	0.006		
Frailty (yes vs. no)	2.43 (0.97–6.07)	0.06			2.82 (1.11–7.18)	0.03	2.69 (0.88–8.17)	0.08^*^
Boredom (yes vs. no)	1.93 (0.79–4.71)	0.15			3.78 (1.46–9.77)	0.006	4.06 (1.63–12.11)	0.01^***^
Leisure time activities (low vs. high)	2.00 (0.81–4.93)	0.13			1.89 (0.76–4.72)	0.17		
Wants more friends (yes vs. no)	1.49 (0.61–3.63)	0.39			2.52 (0.97–6.53)	0.06		
Wants to participate in more activities (yes vs. no)	1.55 (0.64–3.77)	0.33			0.69 (0.28–1.711)	0.43		
Livable community (low vs. high)	2.52 (0.80–7.92)	0.11			4.70 (1.42–15.52)	0.01	3.35 (0.81–13.87)	0.09^*^
Knowledge about community services (low vs. high)	3.09 (1.22–7.84)	0.02			5.90 (2.06–16.91)	0.001	4.61 (1.42–15.02)	0.01^***^
Interest in intergenerational activities (yes vs. no)	0.39 (0.14–1.13)	0.08	0.19 (0.05–0.69)	0.01^***^	1.93 (0.72–5.16)	0.19		

OR, Odds Ratio; CI, 95% Confidence Interval; *Statistical significance at the 0.10 level; **Statistical significance at the 0.05 level; ***Statistical significance at the 0.01 level.

#### 5.3.1 Social isolation

In the final adjusted model, we identified three significant predictors of social isolation. Participants who reported resource-related barriers to aging-in-place were 6.56 times more likely than those with no barriers to experience social isolation [95% CI (2.00–21.57), *p* = 0.002]. Participants living in the county for less than 5 years were 3.35 times more likely to experience social isolation compared to those residing in the county for longer term [95% CI (1.04–10.81), *p* = 0.04]. In contrast, the odds of social isolation were lower among older adults interested in intergenerational activities [Adjusted OR (AOR) = 0.19, 95% CI (0.05–0.69), *p* = 0.01] compared to those who were not interested in participating in activities with youth or young adults. The final adjusted model demonstrated good predictive ability (c-statistic, 0.746) (54, 55).

#### 5.3.2 Loneliness

Four significant risk factors were identified via stepwise logistic regression. Participants who were frail were 2.69 times more likely than those without frailty to experience loneliness [95% CI (0.88–8.17), *p* = 0.08]. Reporting boredom quadrupled the likelihood of loneliness compared to those without boredom [AOR: 4.06, 95% CI (1.63–12.11), *p* = 0.01]. Likewise, participants with limited knowledge about community services were 4.61 times more likely than those with more knowledge to report being lonely [95% CI (1.42–15.02), *p* = 0.01]. Finally, older adults who rated their community livability as low were 3.35 times more likely to experience loneliness compared to those who rated their community livability as high [95% CI (0.81–13.87), *p* = 0.09]. The final adjusted model demonstrated good predictive ability (c-statistic, 0.811) (54, 55).

## 6 Discussion

This community needs assessment highlights a successful grassroots initiative to expand access to data about SI/L. The needs assessment also identifies factors associated with older adults’ SI/L, such as living in the county for less than 5 years, reporting resource-related barriers to aging-in-place, boredom, limited knowledge about community services, frailty, and perceived community livability. Local leaders and community stakeholders can use these data to guide policy and practice recommendations to support aging-in-place in the county.

### 6.1 Strength of the approach in a rural context

The approach merits several strengths in the rural context. First, the community needs assessment builds on existing efforts in the community to address the unmet needs of rural older adults. By proactively engaging local community leaders, many of whom are 65 years or older, in the process, our approach champions grassroots engagement and ownership of the initiative (10). Second, our approach leverages local capacity, resources, and readiness for change. Rural nonprofits often face financial, geographic, and staffing challenges that limit their reach and impact on rural older adults (10, 32). A network approach mobilizes multi-sectoral stakeholders and creates the necessary infrastructure to implement change initiatives to address the health and social needs of rural older adults (10). Last, our approach provides community leaders with access to local data to drive policies and the development of initiatives to better support rural older adults (10, 11). In national and statewide assessments, data on rural populations are often suppressed due to low response rates and small sample sizes. Grassroots initiatives have the advantage of having access to hard-to-reach populations, such as rural older adults, to aid networks and coalitions in making informed decisions for their communities.

### 6.2 Tailored strategies to address SI/L

A multifaceted approach is needed to address SI/L in this rural community (3). Findings from the needs assessment underscore the importance of addressing the built environment features to enhance access to healthcare services, transportation, broadband, information, and social engagement and volunteer opportunities (3, 56). Additionally, volunteer-led and home-based initiatives that enhance social contact via non-digital means are promising, low-cost approaches that this community can employ to reduce SI/L among rural older adults who have mobility issues, limited income, or lack access to transportation (57, 58).

The findings also highlight the significance of considering the length of residence in this community as a critical factor in understanding social isolation among older adults. Specifically, it reveals that newcomers to the area (i.e., those who have lived in the community for less than 5 years) are at a higher risk of experiencing social isolation. Similar findings have been reported elsewhere (59). This finding underscores the need for the development and implementation of initiatives, such as orientation programs aimed at healthcare staff and community organizations to facilitate a deeper understanding of “what matters” to older adults across various domains, including social, psychosocial, and spiritual determinants of their health and well-being. Orientation programs (60) can equip individuals with the necessary knowledge and tools to engage with older adults in a holistic manner, considering their individual preferences, values, and priorities. Such programs can also assist older adults with navigating the available resources and services in the community. Identifying community engagement opportunities and connecting older adults with those resources can help create a supportive environment that promotes social interactions, community engagement, emotional well-being, and a sense of purpose (3, 31). By incorporating the principles of such person-centered care, these programs may have potential to help professionals develop a comprehensive understanding of each older adult’s unique social support system and psychosocial needs (31).

### 6.3 Practical implications

By investing to build an ecosystem of support, rural communities can address the evolving health and social needs of older residents (60). The American Association of Retired Persons (AARP) Network of Age-Friendly Communities can guide local efforts. The age-friendly community movement builds on the World Health Organization’s eight domains of livability framework which was developed to guide communities in becoming more age-friendly, livable, and connected (61, 62). Despite having a growing proportion of older adults in Central Appalachia (22), age-friendly initiatives have been slow to emerge in the region (63).

Since implementing the needs assessment described in this article, efforts are now underway to work with the AARP State Office to obtain the designation as an Age-Friendly county. Network members will build upon the findings from the needs assessment and conduct listening sessions with the community. The goal is to explore key issues on a deeper level, assess community readiness for change, and identify leverage points and opportunities for improvement. By using a data-driven approach and guidance from evidence-based strategies, network members can tailor efforts to the needs of their community members. Other rural communities can learn practical lessons from this needs assessment to expand local efforts to promote healthy aging.

## 7 Limitations

Our project has several limitations including a small sample size and lack of generalizability of the findings since the data were derived from older adults in a single county. In addition, purposive recruitment and the characteristics of participants (e.g., female, unmarried/divorced, living alone) may partially explain the high percentage of social isolation in this population. We acknowledge the influence of employment on SI/L issues. Employment was not included in the analysis because residents who completed the needs assessment were not employed. Gender was captured in a binary way (male or female) and may not capture the gender diversity of participants. Lastly, a small sample size resulted in a lower precision during data analysis (e.g., wide confidence intervals).

## 8 Conclusion

Overall, this community needs assessment underscores the urgency of addressing SI/L among rural older adults in Central Appalachia and the importance of localized data to inform program development for this population. By implementing data-informed strategies and policies, rural communities can work toward promoting age-friendly initiatives to address SI/L among their older residents.

## Data Availability

The datasets presented in this article are not readily available because researchers are using the dataset to develop other manuscripts. Requests to access the datasets should be directed to Jodi Southerland, southerlanjl@etsu.edu.
